# Chemical Composition and Antimicrobial Activity of *Laurus nobilis* L. Essential Oils from Bulgaria

**DOI:** 10.3390/molecules24040804

**Published:** 2019-02-22

**Authors:** Hafize Fidan, Galina Stefanova, Iliana Kostova, Stanko Stankov, Stanka Damyanova, Albena Stoyanova, Valtcho D. Zheljazkov

**Affiliations:** 1 Department of Catering and Tourism, University of Food Technologies, 4000 Plovdiv, Bulgaria; docstankov@gmail.com (S.S.); aastst@abv.bg (A.S.); 2 Lotos Expert OOD, 4000 Plovdiv, Bulgaria; gpld@abv.bg; 3 Department of Biotechnology and Food Technology, “Angel Kanchev” University of Russe, Razgrad Branch, 7200 Razgrad, Bulgaria; ikostova@uni-ruse.bg (I.K.); sdamianova@uni-ruse.bg (S.D.); 4 Crop and Soil Science Department, Oregon State University, Corvallis, OR 97331, USA; valtcho.jeliazkov@oregonstate.edu

**Keywords:** sweet bay, volatile oils, chemical characterization, antimicrobial activity

## Abstract

Laurel, *Laurus nobilis* L. is an evergreen plant belonging to the Lauraceae family, native to Southern Europe and the Mediterranean area. This is the first report on the composition and bioactivity of laurel essential oil (EO) from Bulgaria. The oil yield was 0.78%, 0.80%, and 3.25% in the fruits, twigs, and leaves, respectively. The main constituents in the fruit EO were 1,8-cineole (33.3%), *α*-terpinyl acetate (10.3%), *α*-pinene (11.0%), *β*-elemene (7.5%), sabinene (6.3%), *β*-phellandrene (5.2%), bornyl acetate (4.4%), and camphene (4.3%); those in the twig EO were 1,8-cineole (48.5%), *α*-terpinyl acetate (13.1%), methyl eugenol (6.6%), *β*-linalool (3.8%), *β*-pinene (3.4%), sabinene (3.3%) and terpinene-4-ol (3.3%); and the ones in the leaf EO were 1,8-cineole (41.0%), *α*-terpinyl acetate (14.4%), sabinene (8.8%), methyl eugenole (6.0%), *β*-linalool (4.9%), and *α*-terpineol (3.1%). The antibacterial and antifungal properties of laurel EOs were examined according to the agar well diffusion method. The leaf EO showed antibacterial and antifungal activities against almost all strains of the microorganisms tested, whereas the twig EO was only able to inhibit *Staphylococcus aureus*. *Pseudomonas aeruginosa* ATCC 9027 and *Escherichia coli* ATCC 8739 were the bacterial strains that showed the highest resistance to the laurel EO. The results can benefit the EO industry and biopesticide development.

## 1. Introduction

Laurel or sweet bay (*Laurus nobilis* L.) of the Lauraceae family is a plant native to the southern Mediterranean region. It is grown as high-value spice crop in that region, and as an ornamental plant throughout Europe and America. It also grows as an escapee, naturalized in eastern Bulgaria, along the Black Sea coast.

Aqueous extracts of laurel fruits and leaves have been used in herbal medicine as an astringent agent and for the treatment of several neurological, dermatological, and urological disorders [1]. In addition, laurel essential oil (EO) is currently used in folk medicines for the treatment of different health problems, such as rheumatism and dermatitis [1,2].

Phytochemical analyses have shown the presence of compounds of volatile and non-volatile oils, flavonoids, tannins, sesquiterpenic alcohols, alkaloids, minerals, and vitamins [2,3].

The laurel EO yield and composition were shown to be influenced by various factors, such as growth environment, harvest season, plant parts, extraction method, and others. The EO content (yield) of the laurel fruits varied within a large range, 0.60–4.30% [2,3,4,5,6,7,8,9,10], and the EO content of the leaves also varied widely, from 0.5 to 4.3% [2,11,12,13,14,15,16,17,18,19,20,21,22,23,24,25,26].

The main compounds of the fruit EOs in previous studies were 1,8-cineole (8.10–48.0%), *α*-terpinyl acetate (3.67–10.4%), sabinene (4.49–11.4%), *α*-phellandrene, eugenol, methyl eugenol, *α*-pinene; *β*-ocimene, *β*-pinene, etc., (3.91–12.8%) [2,5,6,7,8,9,10].

The leaf EO was found to be rich in 1,8-cineole (30–70%), linalool (0.9–26.9%), *α*-terpinyl acetate (4.50–25.7%), *α*-pinene, *β*-pinene, sabinene, *α*-terpineol, terpineol-4, etc. [11,12,13,14,15,16,17,18,19,20,21,22,23,24,25,26]. The growing interest in natural products, such as EOs, and the inclusion of plant extracts in various cosmetic products is a prerequisite for an in-depth analysis of the chemical composition of laurel genotypes from various regions. According to some studies [27,28], certain EO constituents may cause allergic reactions when included in cosmetic products.

The laurel EOs have demonstrated antimicrobial [14,15,16,17,18,24,26,29,30,31,32,33], antioxidant [12,15,16,20,21,23,26,32,33], and pharmacological properties [13,33]. Because of its biological activity, laurel leaf EO could be considered a natural supplement or antioxidant in cosmetics [1,34] and medicine [1,8].

In Bulgaria, laurel is found mainly as an ornamental plant or escapee in the wild in the southernmost regions. The majority of the laurel genotype pool in Bulgaria was probably introduced from Greece after the Second World War [35]. However, some may have been transferred from Middle Eastern countries during the 15th to 18th century, or the Ottoman period. Generally, laurel fruits are not harvested and, therefore, not used although they have a potential to provide an EO with unique characteristics and other novel bioactivities. The leaves have been used as a spice and preservative in the food industry for making various products, as well as in folk medicine. According to Georgiev and Lazarov [36], the laurel trees found in Bulgaria had a sufficient EO content, which was higher in plants growing in the warmer parts of the country. The leaves of the middle part of the shoots had a higher EO content (1.9–3.35%). The EO in the twigs (less than 0.4%) allowed them to be used, together with the waste from the processing of the leaves (crushed and non-standard leaves), as a raw material for EO production. However, the latter authors did not analyze the EO composition. 

There has been a growing interest in biologically active substances from non-traditional and underexplored plant species. Bulgaria is one of the largest EO producers in Europe; its distillation facilities and network could easily accommodate a novel EO from naturalized plants produced locally. Despite the interest demonstrated by the industry, there is no previous study on the composition and antimicrobial activity of EOs of laurel found in Bulgaria.

This study aimed to determine the chemical composition, antibacterial, and antifungal activity of the EOs from different parts of laurel grown in Bulgaria as a possible source of constituents for use in perfumery, cosmetics, and pharmaceutical products.

## 2. Results

In this study, the EO content (yield) was 0.78% ± 0.01% in the fruits, 0.80% ± 0.01% in the twigs, and 3.25% ± 0.03% in the leaves. 

### 2.1. Chemical Composition

The chemical composition of the EOs from laurel leaves, twigs, and fruits is listed in Table 1. The laurel EO was light yellow and had a specific odor. In the fruit EO of this study, 38 constituents representing 99.3% of the total content were identified. Twelve of the constituents were in concentrations over 1% of the EO. The main constituents in the fruit EO (above 3%) were 1,8-cineole (33.3%), α-terpinyl acetate (10.3%), *α*-pinene (11.0%), *β*-elemene (7.45%), sabinene (6.30%), *β*-phellandrene (5.2%), bornyl acetate (4.38%), and camphene (4.3%).

Thirty-seven volatile constituents, representing 98.8% of the total composition were identified in the twig oil, 12 of them exceeding 1%. The most abundant constituents found in the twig EO (above 3%) were 1,8-cineole (48.5%), *α*-terpinyl acetate (13.1%), methyl eugenol (6.6%), *β*-linalool (3.8%), *β* -pinene (3.4%), sabinene (3.3%), and terpinene-4-ol (3.3%).

Results show that 40 constituents representing 98.93% of the total content were identified in the leaf EO, 11 of them being above 1%. The main constituents in the leaf EO (above 3%) were 1,8-cineole (41.0%), *α*-terpinyl acetate (14.4%), *α*-pinene (2.6%), *β*-elemene (0.78%), sabinene (8.8%), *β*-linalool (4.9%), *α*-terpineol (3.1%), *α* -pinene (2.6%), and terpinene-4-ol (2.4%).

### 2.2. Antimicrobial Activity

The results of the antimicrobial assay are presented in Table 2. The laurel fruit EO in this study showed low inhibitory activity against Gram-positive bacteria *Staphylococcus aureus* and *Kocuria rhizophila*, Gram-negative bacterium *Salmonella abony*, yeast *Saccharomyces cerevisiae,* and fungus *Aspergillus brasiliensis*. However, the fruit EO did not show inhibitory activity against Gram- negative bacteria *Esсherichia coli* and *Pseudomonas aeruginosa*.

The laurel leaf EO in this study possessed low antimicrobial potential against Gram-positive bacteria *Staphylococcus aureus*, *Kocuria rhizophila,* and *Bacillus subtilis*, Gram-negative bacterium *Salmonella abony*, and fungus *Aspergillus brasiliensis*. *Candida albicans* was sensitive, while *Saccharomyces cerevisiae* showed high sensitivity to the inhibitory effect of the leaf EO. *Escherichia coli* and *Pseudomonas aeruginosa* were resistant to the inhibitory activity of the leaf EO. The laurel twigs EO showed weak action against *Staphylococcus aureus* but not against the other test microorganisms in this study.

## 3. Discussion

The differences between the Bulgarian laurel EO composition in this study and that from other countries reported in the literature are probably due to the different genotypes, climatic conditions in the respective locality where the plants were grown, and also to the plant parts processed and extracted.

The concentration of 1,8-cineole in the fruit EO in this study was similar to the respective concentrations in the EO from Jordan (29.8%) [3], Croatia (32.3%) [7], and Lebanon (31.8%) [5]. Furthermore, the 1,8-cineole concentration in the fruit EO in this study was higher than those reported from Turkey (9.5%) [2], Lebanon (9.4%) [4], and from another area in Lebanon (17.6%) [5]. However, the 1,8-cineole concentration in this study was lower than those in reports from Iran (40.5–46.7%) [8], and from a third study in Lebanon (48.0%) [5].

The observed high concentration of 1,8-cineole in the twig EO in this study was the basis for suggesting that in the industrial production of laurel EO, both the leaves and the twigs should be used if a high 1,8-cineole concentration was desirable.

Most abundant leaf EO constituents were: 1,8-cineole (41.0%) and *α*-terpinyl acetate (14.4%), which were comparable to the respective values reported previously [31]. The 1,8-cineole concentration in the leaf EO of *L. nobilis* from Bulgaria was similar to that in some previous reports [31].

The differences in the quantitative and qualitative composition of the EOs, and in their composition determined in this and previous reports could be due to a number of factors, such as the collection location, soil characteristics, climatic conditions, harvest time, possible differences in the plant genotypes, postharvest processing, and the EO extraction method. 

In this study, the oxygenated monoterpenes and monoterpene hydrocarbons were the dominant groups of chemical constituents in the EOs from the three plant parts, followed by phenyl propanoids. 

Fruit and twig EO have a lower content of the linalool and eugenol allergens than that of leaf EO. The European Cosmetic Directive prohibits the use of these allergens unless they are a natural constituent of plant EO or other natural flavoring (as in the case of laurel EO). These allergens must not exceed the permissible concentration of 0.01% in shower gels and rinse-off products and must not be higher than 0.001% in body oils, massage oils, and creams [37]. Due to their opulent chemical composition and characterization, the inclusion of oils in different cosmetic products will be a subject of our next research.

**Figure 1:**
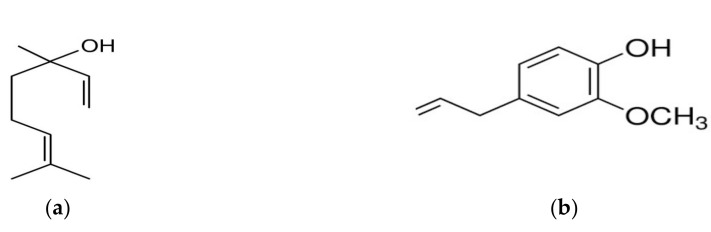
Chemical structures of (**a**) linalool and (**b**) eugenol.

Linalool (Figure 1) is a monoterpene alcohol which occurs as one of its enantiomers in many EOs, where it is often the main constituent. For example, (−)-linalool occurs at a concentration of 80 to 85% in *Cinnamomnum camphora* oil, and rosewood oil contains around 80%. (+)-Linalool makes up 60 to 70% of coriander oil. Eugenol (Figure 1) belongs to the chemical group of phenyl propanoids and is the main constituent of several EOs; e.g., clove oil and cinnamon leaf oil may contain up to 90% eugenol [38].

Laurel EO has been reported to inhibit a broad spectrum of microorganisms. Overall, the results from this study are in line with previous literature reports [13,39,40,41]. Laurel EO has shown significant antibacterial properties and greater effectiveness against some microorganisms than tetracycline antibiotics [39]. Our results are in good agreement with the ones reported by Caputo et al. [11]. A possible explanation for the antibacterial activity of laurel EO is the fact that plant EOs disrupt cellular membranes and increase membrane permeability; they may alter membrane-embedded proteins and subsequently disrupt membrane transport. It was previously demonstrated that terpenes are the constituents responsible for the antibacterial activity of laurel EO [40].

There are several different methods for testing the antimicrobial activity of plants and their constituents, which may significantly influence the observed levels of inhibition. Additionally, various other factors, such as seasonality, variability the plant material, and the EO composition within a plant species, may cause differences in the antimicrobial activity outcomes. This study showed that laurel EOs were effective against all tested Gram-positive bacterial strains, while two of the Gram-negatives were completely resistant to the tested EOs. Gram-positive bacteria are generally more susceptible to the action of the oils compared with the Gram-negative ones. This is due to the presence of an additional outer membrane in Gram-negative bacteria, which may better protect the cytoplasmic membrane from the antimicrobial compounds, such as EOs [42]. The main constituent of laurel EO in this and in some previous studies was 1,8-cineole, that has shown antimicrobial activity against several microorganisms [13,29]. Each EO included an admixture of a number of constituents that may have contributed to the extended spectrum of antimicrobial activity. No assays with pure 1,8-cineole were conducted in this study though. The inhibition strength of laurel EO on microbial growth in this study was probably due to the synergistic or antagonistic effect of 1,8-cineole with oxygenated terpenes of the oil [29,43,44,45].

**Figure 2:**
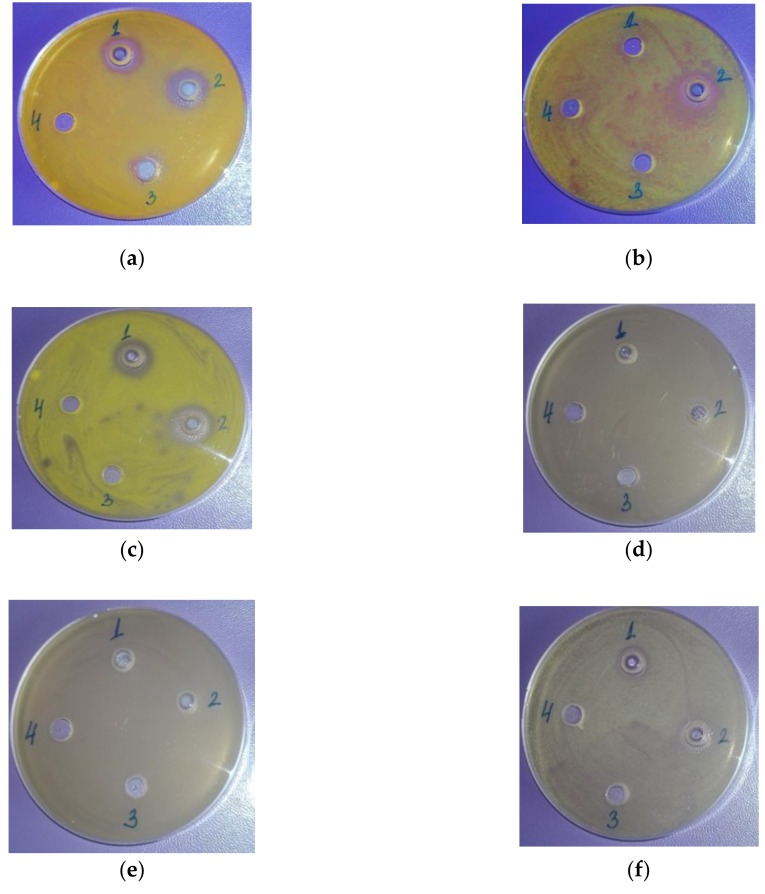
Antimicrobial activity of laurel (*Laurus nobilis* L.): (1) fruit EO; (2) leaf EO; (3) twig EO (solution in nonpolar solvent; (4) nonpolar solvent against: (**a**) *Staphylococcus aureus*; (**b**) *Bacillus subtilis;* (**c**) *Kocuria rhizophila*; (**d**) *Escherichia coli;* (**e**) *Pseudomonas aeruginosa*; (**f**) *Salmonela abony*

The inhibitory effects of laurel fruit, leaf and twig EOs against selected target microorganisms are shown in Figure 2.

## 4. Materials and Methods 

### 4.1. Plant Material 

The laurel fruit, twigs, and leaves were harvested from wild-grown trees in 2018 in the vicinity of the town of Nesebar, eastern Bulgaria, along the Black See coast, a region characterized by a temperate continental climate. The plant species was identified as *Laurus nobilis* L. by the Department of Botany and Methods of Biology Teaching, Faculty of Biology, Paisii Hilendarski University of Plovdiv in Plovdiv, Bulgaria, according to the morphological features of the plant described in the European Pharmacopoeia and the Flora Europaea.

The moisture of the fruit (17.05% ± 0.16), twigs (10.37%% ± 0.09) and leaves (7.83%% ± 0.06) was determined by drying up to constant weight at 105 °C [46].

### 4.2. Isolation of the Essential Oil

The air-dried fruits were ground in a laboratory mill to a size of 0.7 to 1 cm, and the twigs and leaves were cut to a size of 1 cm. The EO was isolated by hydrodistillation for 3 h in a laboratory glass apparatus of the British Pharmacopoeia modified by Balinova and Diakov [47]. The oil obtained was dried over anhydrous sodium sulfate and stored in tightly closed dark vials at 4 °C until analysis. The EO yields are represented on absolute dry weight basis.

### 4.3. Gas Chromatographic (GC) Mass Spectroscopy (MS) Analyses of the Essential Oil

The gas chromatographic (GC) analyses of all samples of laurel EO from Bulgaria were performed using a GC Agilent 7890A, an HP-5 ms column (30 m × 250 µm × 0.25 µm), temperature: 35 °C/3 min, 5 °C/min to 250 °C for 3 min, total: 49 min; helium as a carrier gas at 1mL/min constant speed, and 30:1 split ratio. The GC/MS analysis was carried out on an Agilent 5975C mass spectrometer, using helium as a carrier gas, and the same column and temperature as in the GC analysis. The identification of chemical compounds was made by comparison to their relative retention time and library data. The identified constituents were arranged in order of retention time and quantity in percentage.

### 4.4. Antimicrobial Activity of the Essential Oil

The antibacterial activity of laurel fruit, twig, and leaf EOs was tested against test microorganisms provided by the National Bank for Industrial Microorganisms and Cell Cultures in Sofia, Bulgaria: Gram-positive bacteria: *Staphylococcus aureus* ATCC 6538, *Bacillus subtilis* ATCC 6633, *Kocuria rhizophila* ATCC 9341; Gram-negative bacteria: *Escherichia coli* ATCC 8739, *Pseudomonas aeruginosa* ATCC 9027, *Salmonella abony* NTCC 6017; yeast: *Saccharomyces cerevisiae* ATCC 2601, *Candida albicans* ATCC 10231; and fungal strain: *Aspergillus brasiliensis* ATCC 16404.

The antimicrobial activity was determined by the agar well diffusion method with a well size of 8 mm. The growth media were Tryptic soy agar (Merck) for the tested bacterial strains and Sabouraud–Dextrose–Agar (Merck) for the yeast and fungi. The media were inoculated with a 24-h suspension of the bacterial species with a density of approximately 10^7^ cfu (colony forming units)/mL (turbidity: 0.5 McFarland standards). Media melted and cooled to 50 °C were inoculated with the tested microorganisms and then equally dispensed into Petry dishes. Next, a hole with a diameter of 8 mm was punched aseptically with a sterile cork borer, and a volume (50 µL) of the antimicrobial agent was introduced into the well. After that, the agar plates were incubated at 37 °C or 28 °C for 24 or 72 h according to the microbial species. After cultivation, the distinct zone of growth inhibition around the wells was measured using a digital caliper. The diameter of the zones, including the diameter of the well, was recorded in mm, for instance, up to 15 mm the microbial culture was poorly sensitive, from 15 to 25 mm it was considered sensitive, and over 25 mm it was considered very sensitive. The tests were performed in parallel with solvent controls [48].

### 4.5. Statistics

All the analyses and measurements were done in triplicate. The results are presented as the mean value of the individual measurements with the corresponding standard deviation (SD).

## 5. Conclusions

This is the first study on the EO composition and bioactivity of laurel fruits, leaves, and twigs from Bulgaria. The laurel EOs from Bulgaria was characterized by a higher content of 1,8-cineole and α-terpinyl acetate with a characteristic odor. The oils produced demonstrated antimicrobial activity against some of the highly susceptible strains of pathogenic and spoilage bacteria and yeasts. Laurel fruit and leaves, along with the branches that carry them, can be used as a non-traditional material for the production of EOs to be used as an additive in the cosmetic industry. Currently, the studies on the application of laurel EOs in cosmetic and food products are in progress.

## Figures and Tables

**Table 1:** Chemical composition of the laurel (*Laurus nobilis* L.) fruits, twigs, and leaves essential oil.

№	Compounds	RI	Content, %		
			Fruits	Twigs	Leaves
1	*α*-Thujene	931	nd *	0.29 ± 0.00	0.32 ± 0.00
2	*α*-Pinene	939	11.01 ± 0.15	2.94 ± 0.03	2.56 ± 0.03
3	Camphene	954	4.33 ± 0.05	0.30 ± 0.00	0.18 ± 0.00
4	Sabinene	971	6.30 ± 0.07	3.33 ± 0.07	8.82 ± 0.13
5	*β*-Pinene	979	0.28 ± 0.00	3.44 ± 0.07	2.45 ± 0.06
6	*β*-Myrcene	991	0.34 ± 0.00	0.19 ± 0.00	0.31 ± 0.00
7	*α*-Phellandrene	1003	5.18 ± 0.06	0.38 ± 0.00	1.01 ± 0.02
8	*α*-Terpinene	1014	0.22 ± 0.00	0.89 ± 0.00	0.52 ± 0.00
9	*p*-Cymene	1020	nd	1.00 ± 0.02	0.18 ± 0.00
10	Limonene	1029	2.25 ± 0.04	1.68 ± 0.03	0.04 ± 0.00
11	1,8-cineole	1032	33.33 ± 0.70	48.53 ± 0.75	41.02 ± 0.71
12	*cis-β*-ocimene	1046	0.16 ± 0.00	nd	nd
13	*trans-β*-ocimene	1050	0.72 ± 0.00	nd	nd
14	*γ*-Terpinene	1055	0.44 ± 0.00	1.35 ± 0.03	0.99 ± 0.02
15	*cis*-Sabinene hydrate	1065	nd	nd	0.62 ± 0.00
16	*β*-Linalool	1096	2.16 ± 0.06	3.80 ± 0.07	4.92 ± 0.08
17	Terpinene-*4*-ol	1179	0.85 ± 0.00	3.25 ± 0.07	2.35 ± 0.04
18	*α*-Terpineol	1189	1.55 ± 0.04	1.73 ± 0.05	3.11 ± 0.06
19	Bornyl acetate	1286	4.38 ± 0.08	0.52 ± 0.00	0.65 ± 0.00
20	*α*-Terpinyl acetate	1333	10.30 ± 0.30	13.09 ± 0.33	14.44 ± 0.35
21	Thymol	1336	0.20 ± 0.00	0.70 ± 0.00	0.15 ± 0.00
22	Eugenol	1363	0.21 ± 0.00	0.33 ± 0.00	1.47 ± 0.02
23	*β*-Elemene	1390	7.45 ± 0.07	0.25 ± 0.00	0.78 ± 0.00
24	Methyleugenol	1402	1.58 ± 0.04	6.62 ± 0.06	6.03 ± 0.06
25	*β*-Caryophyllene	1429	0.51 ± 0.00	0.35 ± 0.00	0.32 ± 0.00
26	Germacrene D	1484	nd	nd	0.25 ± 0.00
27	Bicyclogermacrene	1501	nd	nd	0.16 ± 0.00
28	Caryophyllene oxide	1574	0.61 ± 0.00	0.41 ± 0.00	0.34 ± 0.00
29	Ledol	1602	0.31 ± 0.00	0.27 ± 0.00	0.39 ± 0.00
30	(−)-Spathulenol	1619	0.25 ± 0.00	0.21 ± 0.00	0.31 ± 0.00
31	*τ*-Cadinol	1628	0.44 ± 0.00	0.38 ± 0.00	0.55 ± 0.00
32	*β*-Eudesmol	1642	0.37 ± 0.00	0.32 ± 0.00	0.47 ± 0.00
34	Cedren-13-ol acetate<8->	1788	0.97 ± 0.00	nd	nd
34	*n*-Heneicosane	2100	0.19 ± 0.00	0.16 ± 0.00	0.24 ± 0.00
35	Phytol	2105	0.21 ± 0.00	0.18 ± 0.00	0.26 ± 0.00
36	*n*-Docosane	2200	0.21 ± 0.00	0.18 ± 0.00	0.26 ± 0.00
37	*n*-Tricosane	2300	0.19 ± 0.00	0.17 ± 0.00	0.23 ± 0.00
38	*n*-Tetracosane	2400	0.16 ± 0.00	0.15 ± 0.00	0.20 ± 0.00
39	*n*-Pentacosane	2500	0.24 ± 0.00	0.21 ± 0.00	0.30 ± 0.00
40	*n*-Hexacosane	2600	0.39 ± 0.00	0.34 ± 0.00	0.49 ± 0.00
41	*n*-Heptacosane	2700	0.33 ± 0.00	0.28 ± 0.00	0.40 ± 0.00
42	*n*-Octacosane	2800	0.26 ± 0.00	0.23 ± 0.00	0.33 ± 0.00
43	Squalene	2817	0.41 ± 0.00	0.35 ± 0.00	0.51 ± 0.00
Total identified compounds, %			99.29	98.80	98.93
Hydrocarbons, %			1.98	1.74	2.48
Monoterpene hydrocarbons, %			31.45	14.97	17.38
Oxygen monoterpenes, %			52.95	71.78	67.84
Sesquiterpene hydrocarbons, %			8.02	0.61	1.53
Oxygen sesquiterpenes, %			2.97	1.61	2.08
Phenyl propanoids, %			2.00	8.76	7.91
Diterpenes, %			0.21	0.18	0.26
Triterpenes, %			0.41	0.35	0.52

* Not detected.

**Table 2:** Antimicrobial activity of laurel (*Laurus nobilis* L.) fruits, twigs, and leaves essential oil (EO).

Test Microorganisms	Inhibition Zone, mm		
	Fruit EO	Twigs EO	Leaves EO
*Staphylococcus aureus* ATCC 6538	12.9 ± 0.02	11.4 ± 0.05	15.1 ± 0.02
*Bacillus subtilis* ATCC 6633	8.0 ± 0.00	8.0 ± 0.00	13.6 ± 0.05
*Kocuria rhizophila* ATCC 9341	11.6 ± 0.05	8.0 ± 0.00	13.0 ± 0.00
*Escherichia coli* ATCC 8739	8.0 ± 0.00	8.0 ± 0.00	8.0 ± 0.00
*Pseudomonas aeruginosa* ATCC 9027	8.0 ± 0.00	8.0 ± 0.00	8.0 ± 0.00
*Salmonela abony* NCTC 6017	11.3 ± 0.02	8.0 ± 0.00	12.2 ± 0.04
*Candida albicans* ATCC 10231	8.0 ± 0.00	8.0 ± 0.00	16.4 ± 0.02
*Saccharomyces cerevisiae* ATCC 2601	15.7 ± 0.04	8.0 ± 0.00	33.3 ± 0.00
*Aspergillus brasiliensis* ATCC 16404	10.8 ± 0.02	8.0 ± 0.00	14.8 ± 0.05
